# 医患共同决策诊疗模式在胸部肿瘤患者中的应用进展

**DOI:** 10.3779/j.issn.1009-3419.2024.101.04

**Published:** 2024-02-20

**Authors:** Weihao CHEN, Mengni ZHANG, Cheng SHEN

**Affiliations:** ^1^610041 成都，四川大学华西医院临床医学院（陈伟皓）; ^1^West China School of Medicine, West China Hospital, Sichuan University, Chengdu 610041, China; ^2^610041 成都，病理科（张孟尼）; ^2^Department of Pathology, West China Hospital, Sichuan University, Chengdu 610041, China; ^3^610041 成都，胸外科（沈诚）; ^3^Department of Thoracic Surgery, West China Hospital, Sichuan University, Chengdu 610041, China

**Keywords:** 医患关系, 共同决策, 胸外科, 胸部肿瘤, Doctor-patient relationship, Shared decision making, Department of Thoracic Surgery, Thoracic cancer

## Abstract

医患共同决策（shared decision making, SDM）作为新型的诊疗决策模式，能够有效解决胸部肿瘤诊疗过程中患者的依从性问题，平衡医患双方的地位，逐渐在胸外科临床实践中得到重视和应用。将SDM应用于胸部肿瘤诊疗过程中有利于提升医生的诊疗水平，缓解责任压力；减轻患者的心理压力，提高患者的依从性；提高医信力并减少医患冲突。由于患者自身的医学素养和自主性有限，医患比例失衡导致的诊疗时间紧张，同时由于已有的研究存在样本量有限等原因尚不能证明采用SDM模式对于胸部肿瘤的治疗有明确的增益，推行SDM模式仍面临阻力。未来通过开发辅助决策系统以及提升医生的人文关怀能力将更有利于推动SDM模式在胸外科的实际应用。

## Abstract (en)

As a new diagnosis and treatment decision-making model, shared decision making (SDM) can effectively solve the problem of patient compliance in the diagnosis and treatment of thoracic tumors, balance the status of both doctors and patients, and gradually get attention and application in the clinical practice of thoracic surgery. The application of SDM in the diagnosis and treatment of thoracic tumors is conducive to improve doctors' diagnosis and treatment level and alleviating the pressure of responsibility, reduce patients' psychological pressure and improve patients' compliance and also improve medical trust and reduce doctor-patient conflict. Due to the limited medical literacy and autonomy of patients, the time for diagnosis and treatment is short due to the imbalance of doctor-patient ratio. Meanwhile, due to the limited sample size of existing studies, SDM model cannot be proved to have a clear gain for the treatment of thoracic tumors, and the implementation of SDM model still faces resistance. In the future, the development of auxiliary decision-making system and the improvement of doctors' humanistic care ability will be conducive to promote the practical application of SDM model in thoracic surgery.

医患共同决策（shared decision making, SDM）的理念最初是由Veatch^[1]^于1972年在《革命性时代医学伦理的模型》一文中提出，SDM是指医疗决策过程中，让患者参与到各个环节中，医生向患者阐明各种诊疗方案的利弊，聆听患者表达自身的想法、担忧和需求，进行充分的沟通，在尊重患者选择且促进患者康复的基础上，最终由医患双方做出共同决策^[2]^。近年来由于全民医学素养的提升，越来越多的患者倾向于对自身病情有充足的了解并且希望能参与到治疗方案的决策过程中。本文就SDM在胸外科手术领域的适用性以及在我国的发展前景做一综述。

## 1 SDM的临床应用现状

### 1.1 SDM在临床中的应用

对于SDM应用于临床，当下的研究重点主要分为两部分，一部分是什么样的患者更倾向于选择SDM模式，另一部分是对SDM应用于临床不同领域的实际效果评估，研究的具体方式主要为采用问卷、量表的观察性研究^[2⇓⇓⇓⇓-7]^，对于临床实际是否选择SDM模式提供参考，同时为SDM的临床可行性提供了依据。

在龙杰等^[2]^纳入238例肿瘤专科医院患者的临床研究中，大部分患者在临床实践中认为应当采取合作方式进行决策，对于检查方案，有71.85%的患者认为应当采取合作做决策；对于治疗方案，有57.14%的患者认为应当采取合作做决策；对于恢复方案，有70.17%的患者认为应当采取合作做决策。可以看到，在整个肿瘤的诊疗过程中，大部分患者表示出希望与医生共同决定方案的意愿，随着全民医疗卫生素养的提高，SDM模式在临床实践的推行具有良好的患者基础。与此同时，何依群等^[7]^对泌尿系统肿瘤患者的研究表明文化程度较高、疾病分期较高、年龄相对较小、自费是主动型参与手术决策的影响因素，这提示我们在临床实践中选择具体的决策方式时可以参考患者的个人史进行灵活的判断。除此之外，患者的个人性格可能影响到患者的自主性，临床医生进行选择时对于患者的心理评估也同样是重要的环节^[8]^，只有对患者进行全面综合性评估后合理选择应用SDM模式，才能让这种方式正确发挥出最大的作用。

### 1.2 SDM在胸外科肿瘤患者临床中的应用

肺癌和食管癌在我国仍有较高的发病率。据统计^[9]^，在2022年，中国新诊断出来的肺癌总病例数约为106.06万，相当于每天诊断出2900多例肺癌，约占所有恶性肿瘤的22%，肺癌发病率居于中国恶性肿瘤发病率之首，与此同时，2022年，中国新确诊的食管癌患者总数为22.4万例，死亡病例为18.75万例。随着治疗手段的多样化以及生物分子靶向治疗位点的不断发现，肺癌和食管癌等胸部常见肿瘤已经成为生存期相对较长的慢性疾病，慢性疾病的治疗要注重健康管理涉及到漫长的疗养过程，治疗地点选择医院还是居家，治疗方式选择手术、放疗、化疗还是其他方式，都需要医生与患者及其家属进行全面的沟通后共同做出决策，充分尊重患者的动态自主性。与此同时，在漫长的治疗过程中更需要医生在与患者的全程共同决策中不断坚定患者的治疗信心，及时做好患者的心理疏导工作^[8]^。

外科手术仍是肺癌和食管癌重要的治疗方式^[10]^，其技术早期为开胸手术，创伤较大，虽然随着胸腔镜手术技术的成熟，创伤程度在手术过程中有所改善，但是胸部肿瘤的手术治疗仍然给患者带来较大的心理负担和生理不适。采用SDM模式能够有效解决医患关系的不对称性，让患者了解手术的具体情况，调动患者的主观能动性，在满足治疗要求的前提下尽量减少患者受到的伤害，减轻患者心理痛苦，缓解焦虑抑郁情绪，提高患者的术后自理能力，更有利于疾病的康复^[3]^。

胸部肿瘤患者所处的病情发展期具有阶段性和复杂性，SDM模式能够精准地满足患者各个时期的需求，对于疑似肿瘤的患者、早中期患者、晚期及终末期患者，他们提出的困惑也不尽相同。例如对于疑似肿瘤的患者，医生需要向患者解释肺结节和肺癌的关系，让患者理解需要进一步检查才能明晰病情^[11]^。胸部肿瘤的阶段性和复杂性决定了医生需要通过与患者的沟通交流制定个性化的诊疗方案，提高不同时期患者的治疗效果和生存质量，以免发生医患纠纷和过度医疗的情况。

## 2 SDM在胸外科肿瘤疾病应用中的价值

### 2.1 SDM对胸外科医生的价值

胸部肿瘤患者的情况各异，病情复杂多样，胸外科医生能够通过SDM模式与不同背景的患者进行深入的沟通交流，在临床实例中学习患者的需求，通过不断积累阅历提升自己的专业水平和人文素养，教学相长，为患者提供更加具有人文关怀的诊疗体验^[8]^。同时SDM有利于医生与患者共同承担责任，减轻医生选择手术方案的压力，使医生在必要时敢于尝试合理的创新性的手术方案，更加灵活地面对治疗过程中的挑战。

此外，SDM模式可以帮助医生更好地了解患者的需求和偏好，从而制定更符合患者实际情况的个性化的治疗方案。一方面，胸外科疾病的治疗通常需要患者长期配合，例如手术后的康复和药物治疗，患者的依从性对治疗效果至关重要；另一方面，胸外科疾病涉及到复杂的治疗方案和手术决策，患者的年龄、健康状况、生活方式等因素都会对治疗方案产生影响。通过SDM模式，医生可以根据患者的实际情况和偏好制定个性化的治疗方案，增加患者对治疗的信心，减少治疗过程中的不确定性和焦虑情绪，提高患者对治疗方案的接受度和依从性。

与此同时，SDM模式可以减少医患之间的矛盾和误解。通过与患者进行深入的沟通和决策，医生可以更好地解释治疗方案的利弊和风险，让患者更清晰地了解疾病的病因、发展过程、治疗选择和预后，增强患者对治疗过程的参与感和责任感，促进医生和患者之间的信息共享和沟通，避免因信息不对称而导致的医患矛盾和纠纷。患者参与决策过程也能增强对医生的信任，减少对医疗决策的怀疑和不满情绪，从而建立更良好的医患关系。

### 2.2 SDM对肿瘤患者的价值

在传统的家长式决策模式中，患者被动接受医生的诊疗措施，由于相对短暂的就诊时长，患者从医生处获得的信息有限，同时，患者的自主性没有得到充分的体现，医生所获知的患者的生理、心理情况以及患者的偏好情况也相对有限，患者易通过互联网接受一些错误的信息，对自身疾病情况产生困惑，对医生的诊疗方案产生质疑^[12]^；医生也可能因为缺少沟通而进行一些不必要的检查或治疗，造成资源浪费。有研究^[13]^表明，采用SDM模式既有利于直接改善患者的生活质量，也能够通过中介变量功能锻炼依从性间接促进患者的康复。其原因在于SDM模式一方面确保了胸部肿瘤患者与医生能够充分沟通，共享各方面的信息，及时更新肿瘤的进展状况、患者的身体状况，使患者能够得到更加精准和个性化的诊疗方案^[14]^；另一方面尊重了患者的自主性，在医生充分介绍病情和治疗方案的利弊后，患者对胸部肿瘤的部位、恶性程度、手术的方式和风险等具有整体的了解，在此基础上共同做出的决策更加符合患者的心理预期，自然有更加良好的依从性。随着治疗水平的不断发展，胸部肿瘤已不再是无药可医的绝症，更多的胸部肿瘤患者要经历长期的治疗和康复，基于医学伦理学的自主原则、有利原则、不伤害原则和公正原则，患者应当有权利知晓自身疾病状况，并且基于自主意愿在医生的帮助下决定自己未来的治疗手段和生活方式，通过SDM模式为患者患病后的生活质量保驾护航，更有利于提高患者的满意度^[4]^。

目前在胸外科肿瘤疾病中进行SDM模式的应用实例尚有限，但基于胸部肿瘤的疾病特点与SDM模式的适配性，以及其他肿瘤疾病进行实践的有效性，在胸部肿瘤患者中实行SDM模式具有充足的应用潜力和应用前景。卢世兰等^[15]^对311例肺癌患者的研究表明当今肺癌患者实际的决策参与方式仍以被动型决策参与方式为主。患者选择实际决策参与方式受到家庭经济情况、患者的自主性、患者的心理状态等因素的影响。通过改善患者参与决策过程中的负性体验，不断完善患者参与决策的积极体验，了解患者参与决策的相关需求有利于提高患者参与到决策中的积极性。同时SDM模式在胸部肿瘤患者的诊疗中发挥积极作用需要医生尊重患者的价值观和偏好，评估患者的决策能力，提供胸部肿瘤相关的信息支持，解决患者参与决策过程中遇到的困难，重视患者参与决策过程中的心理变化，及时提供负面情绪的疏导，引导患者积极地看待疾病。同时应该关注家庭方面的经济支持和关怀，帮助患者积极参与到治疗决策中。

### 2.3 SDM对医患关系的价值

采用SDM模式便于患者了解胸部肿瘤相关的医学常识，进而理解医生提出的诊疗方案，一方面提高医信力，维持医生基本的权威性，另一方面也使患者更加信任医生，帮助建立良好的医患关系，减少医患冲突的发生^[16]^。同时SDM模式贯穿胸部肿瘤患者预防、治疗、预后、康复的各个阶段，整个过程中都尊重和反映了患者的个人意愿，患者作为共同决定者要分担治疗过程中的责任和风险，在保障患者权利的同时也维护了医生的正当权益，促进医患关系良性健康发展。

## 3 SDM在胸外科肿瘤疾病临床应用中的问题

虽然SDM模式对于胸部肿瘤患者的诊疗过程具有各方面的益处，但是SDM在胸外科的实际应用仍然存在各方面的问题和阻力亟待解决。首先在支持SDM模式应用于患者是有效的理论研究中，目前大部分的研究均为横向研究，缺乏纵向研究的效果探究^[3]^；同时对用于研究的患者样本的选取过程中，现今的研究多局限于单一科室，缺乏多个科室联合对于SDM模式应用于胸部肿瘤患者的实际效果进行综合性的评估^[17]^。其次在患者的参与意愿方面，由于患者的医学素养有限，不同患者的自主性不同，外界信息对患者的干扰等^[12]^，患者参与诊疗决策的实际情况并不乐观。同时在医生实际应用SDM模式方面，由于我国医患比例的不平衡，划分SDM双方责任主体的相关法律法规不健全，医生很难有充足的时间和精力与患者进行SDM的沟通交流。各方面存在的客观问题限制了医患SDM模式在我国胸外科的实际应用，解决落实过程中的各种阻力需要各方、各机构的通力合作。

## 4 展望

随着人工智能技术的完善和发展，辅助决策系统成为未来完善SDM模式在胸外科的实际应用的有力技术，辅助决策系统能够完整地记录患者的偏好，同时全面展示疾病相关的信息和各种治疗手段的利弊，当前国外的决策辅助系统的资源主要包括加拿大渥太华医院研究所的患者决策辅助系统数据库（https://decisionaid.ohri.ca/index.html）以及美国梅奥诊所医患共同决策资源中心（https://shareddecisions.mayoclinic.org），而国内的辅助决策系统仅局限于有限几种疾病，同时缺少充足的数据支撑。相关研究单位近期设计与实现了规则引擎Drools驱动下的肿瘤临床辅助决策系统，在进行逻辑规则判断推理时，能提高效率与准确率^[18]^，未来在肿瘤相关疾病诊断与治疗方面具有充足的潜力。通过录入临床信息数据完善胸部肿瘤相关的数据库，开发与完善辅助系统能够提升SDM的效率^[19]^，为SDM在胸外科应用提供助益^[12]^。

目前国内对于国外特定专科的辅助决策系统在医疗服务中的应用大多局限于借鉴和套用，由于中西文化差异，例如对疾病、治疗方式、医生权威和家庭观念的态度不尽相同，另外基于我国医疗资源分布不均匀和医保政策推陈出新的现状，国外已有的系统存在“水土不服”的问题。立足于我国的基本医疗国情，建立本土化模型，推出新的评估工具应当考虑以下因素：利用人工智能技术为中国医疗决策提供更精准的辅助，利用大数据分析为我国患者提供个性化的医疗决策，将传统中医理念融入辅助决策系统以适应中西医结合治疗的特色方针。通过开发中国特色的医疗辅助决策系统为我国患者提供更加良好的医疗体验。

与此同时，医务工作人员要敢于尝试应用SDM模式，不能因为医患SDM可能面临的压力而拒绝采取相关诊疗方式，积极学习相关进展并不断提高自身的沟通交流能力^[20]^，掌握灵活应用SDM模式的技巧，在面对阻碍时要不断探索解决的方法^[21]^，将自身的临床经验灵活地用于改进SDM，将经典的SDM模式与胸外科实际情况进行有机结合，不断优化患者的诊疗体验。我们可以借鉴国外相关的SDM案例，制定符合我们自身胸外科SDM的应用原则及各种规章制度，提高医务工作者采用SDM的意识，完善相关保障体系，保证患者的相关权益和诊疗质量，进一步促进胸外科SDM模式的应用与发展。

**Competing interests**

The authors declare that they have no competing interests.
